# Total penectomy as treatment option for solitary penile metastasis in hormone sensitive metastatic prostate cancer (mHSPC): Case report with surgical technique

**DOI:** 10.1016/j.eucr.2024.102899

**Published:** 2024-12-05

**Authors:** M. Vukovic, M. Albijanic, N. Radovic

**Affiliations:** Department of Urology, Clinical Centre of Montenegro, Ljubljanska bb, 81000, Podgorica, Montenegro

**Keywords:** Penile metastasis, Radical penectomy

## Abstract

We present a case of a 66-year-old man with a three-year history of Gleason 10 prostate cancer (PCa), who presented with penile pain, erythema, and induration of the penile shaft. His cancer was treated with androgen deprivation therapy (ADT), radiotherapy, and apalutamide, resulting in PSA reduction; however, a solitary penile lesion persisted, necessitating radical penectomy. At 12 months post-surgery, PSA levels and magnetic resonance imaging findings remained stable, with no signs of metastasis. This case highlights the viability of radical penectomy for solitary penile metastasis in hormone-sensitive metastatic prostate cancer (mHSPC), with potential benefits for symptom control and survival.

## Highlights

• Metastasis of prostate cancer to the penis is extremely rare.
• Systemic treatment is method of choice.
• Radical penectomy with perineostomy might be treatment option when penile metastasis persists.
• Recurrence free survival might exceed 12 months after radical penile surgery.

## 1 Introduction

Prostate cancer metastasizing to the external genitalia is exceedingly rare, with penile metastasis being the most common site. However, few cases are reported, typically managed with metastasis-directed therapy, which generally results in poor prognosis and death within 6–9 months.^1^, ^2^, ^3^ The survival benefits of radical surgery remain inconclusive.

Differential diagnoses for suspected penile metastasis include primary penile malignancies, metastatic spread from the pelvic or rectosigmoid regions, and benign conditions such as Peyronie's disease. Diagnostic clarity is achieved through magnetic resonance imaging (MRI) and biopsy, the latter serving as the gold standard. Treatment decisions consider disease stage, patient condition, and quality of life.^4^ This report presents a case of metastatic hormone-sensitive prostate cancer (mHSPC) treated with radical penectomy and perineostomy, with no evidence of disease progression 18 months after initial detection of penile metastasis and 12 months postoperatively.

## 2 Case presentation

A 66-year-old man with a three-year history of oligometastatic prostate cancer (Gleason score 5 + 5, initial PSA 14 ng/mL) presented with penile pain and induration. His initial diagnosis included two metastatic lesions in the thoracic spine (T10 and T11), which were treated with radical radiotherapy and androgen deprivation therapy (ADT). Eight months prior to presentation, he reported penile pain and hardening of the penile shaft at a routine oncology follow-up. Over time, symptoms worsened, leading to difficulty retracting the foreskin and increased pain on palpation.

On physical examination, a solid, one-centimeter mass was observed on the glans penis and ventral surface of the midshaft, seemingly originating from the glans. Digital rectal examination showed an enlarged, firm, nodular prostate. The remainder of the physical exam was unremarkable, with no palpable lymphadenopathy, spinal tenderness, or neurological deficits. Given the mass's suspicious appearance for squamous cell carcinoma (SCC), a penile biopsy was performed. **Photographic documentation of the primary penile tumor was deemed unnecessary; therefore, no images of the original lesion are available**. Pathology revealed multifocal Gleason 10 prostate cancer involving both the glans and corpora of the penis.

A follow-up MRI of the pelvis and prostate showed no evidence of tumor recurrence or lymphadenopathy. Concurrently, a bone scan identified a solitary osteoblastic lesion in the right tibia, and his PSA was recorded at 1.09 ng/mL. Given these findings suggestive of disease progression, apalutamide was added to his therapy. An excisional biopsy of the tibial lesion, conducted after orthopedic consultation, confirmed prostate cancer origin.

Further workup included prostate MRI, which demonstrated a Prostate Imaging Reporting & Data System (PI-RADS) 5 lesion, mildly enlarged pelvic lymph nodes, and a small sclerotic focus in the left iliac bone. No osseous metastatic disease was observed on the bone scan, and chest CT was normal. After discussion with hematology-oncology, the iliac lesion was considered non-metastatic. Treatment decisions were discussed with the patient, and the consensus was to initiate combined intensity-modulated radiation therapy and ADT with leuprolide acetate. He tolerated the treatment well, with a decrease in PSA over the following two years.

**Fig. 1:**
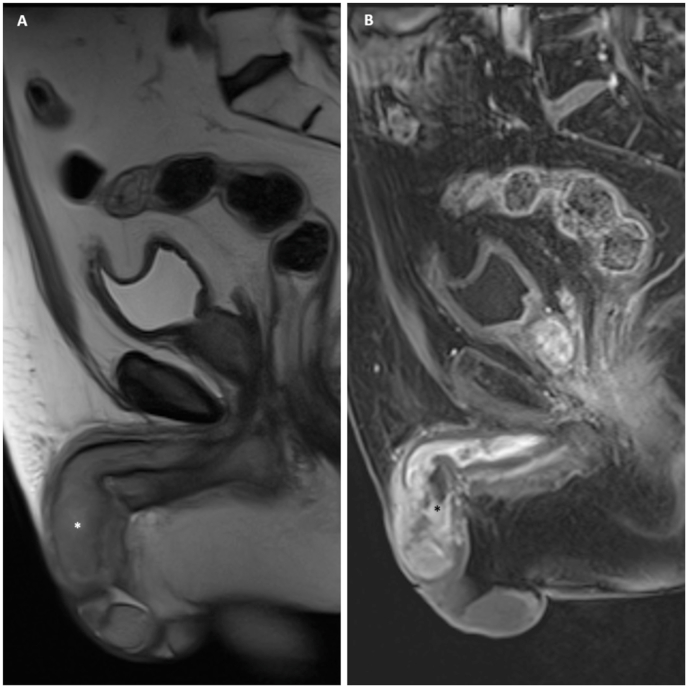
A) Sagittal T2-weighted MR image and B) sagittal post-contrast T1-weighted fat-saturated image showing longitudinal anatomy of penile shaft. Suspected corpora infiltration has been marked as (∗).

Upon ADT completion, PSA levels began to rise, necessitating resumption of hormonal therapy. At this time, he experienced a recurrence of penile pain and urinary symptoms. Repeat physical examination showed a firm, completely indurated penile shaft without exophytic tumors. MRI of the pelvis showed no signs of disease spread, with intact spongious body and urethra (Fig. 1).

**Fig. 2:**
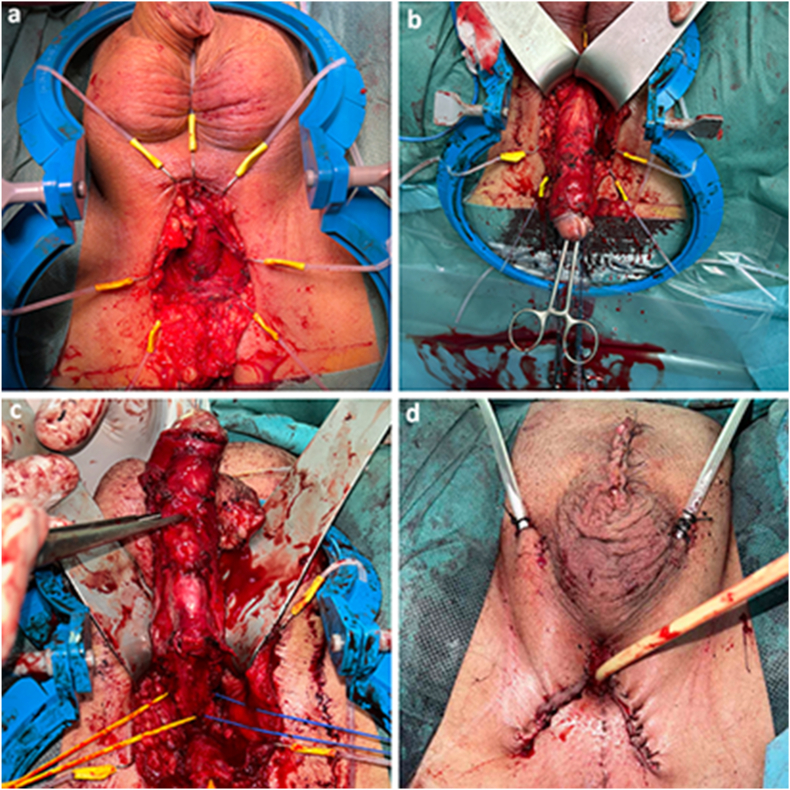
A) Perineal approach for radical penectomy; B) Penile degloving with transperineal displacement; C) Dissection to the level of the crura penis (indicated by yellow and blue markers); D) Final wound appearance following radical penectomy with perineostomy and bilateral orchiectomy.

After a thorough discussion of treatment options, the decision was made to proceed with radical penectomy and perineostomy (Fig. 2). The patient tolerated the surgery well, without postoperative complications. He remained continent, with stable PSA levels and no clinical or radiological evidence of disease progression for 12 months postoperatively.

## 3 Discussion

We present a case of a patient with hormone-sensitive metastatic prostate cancer (mHSPC) who remained metastasis-free for nearly two years following the initial detection of penile metastasis and over 12 months post-radical penectomy, as confirmed through regular PSA monitoring and pelvic MRI. Penile metastasis is an exceptionally rare occurrence, typically presenting in the context of widespread metastatic disease. Common clinical manifestations include penile pain, ulceration, palpable nodules (painful or painless), priapism, urinary retention, dysuria, and hematuria. Our patient initially presented with penile pain and a palpable nodule in the penile shaft. Given his high-volume metastatic disease, systemic treatment was administered initially, which remains the most widely accepted approach for penile metastasis from prostate cancer.

When penile metastasis persists as an isolated metastatic site post-systemic therapy, metastatic-directed therapy (MDT) may become a viable treatment consideration. However, there are no clear guidelines on surgical interventions for symptomatic patients with penile metastasis, and choices range from local excision to partial or total penectomy, depending on patient condition and preferences. MDT has gained attention as a treatment option for oligometastatic hormone-sensitive prostate cancer (mHSPC); however, its impact on overall survival, particularly in cases involving visceral metastasis, remains uncertain 4,5.

According to recent studies, MDT may help maintain PSA control in patients with penile metastasis secondary to prostate cancer, although it is not curative. The decision regarding the most suitable local palliative treatment should be based on the patient's overall condition, the extent of the disease and the intensity of symptoms. Radical penectomy, in this context, may provide a promising palliative outcome—not only by alleviating symptoms such as penile pain but also potentially delaying disease progression when performed in the oligometastatic phase.^1^^,^^6^^,^^7^ A final decision should be made following a thorough discussion with the patient, outlining the potential complications and benefits of radical penile surgery. In our case, the patient opted for radical penectomy and remained clinically and biochemically stable 12 months post-surgery. This outcome contrasts with existing literature, which reports that most patients with penile metastasis succumb to the disease within one year of diagnosis, with an average survival of approximately six months.^4^^,^^6^ This observation underscores the potential advantage of radical over organ-sparing surgery in cases of isolated penile metastasis.

## 4 Conclusion

Penile metastasis from prostate cancer is rare and carries a poor prognosis. Biopsy remains the gold standard for diagnosis and plays a crucial role in guiding management. In the oligometastatic phase, local surgical intervention offers a viable option to improve quality of life. While metastatic-directed therapy (MDT) may have limited impact on overall survival, radical penectomy with perineostomy has the potential to enhance both quality of life and survival outcomes in selected patients.

## CRediT authorship contribution statement

**M. Vukovic:** Writing – review & editing, Writing – original draft, Methodology, Investigation, Formal analysis, Data curation, Conceptualization. **M. Albijanic:** Validation, Supervision, Conceptualization. **N. Radovic:** Methodology, Formal analysis, Data curation.

## Patient consent

Informed consent for patient information to be published in this article was obtained.

## Funding

This research did not receive any specific grant from funding agencies in the public, commercial, or not-for-profit sectors.

## Declaration of competing interest

None.
